# The effect of gadolinium-based contrast agent administration on magnetic resonance fingerprinting-based T_1_ relaxometry in patients with prostate cancer

**DOI:** 10.1038/s41598-020-77331-4

**Published:** 2020-11-24

**Authors:** Nikita Sushentsev, Joshua D. Kaggie, Guido Buonincontri, Rolf F. Schulte, Martin J. Graves, Vincent J. Gnanapragasam, Tristan Barrett

**Affiliations:** 1 Department of Radiology, Addenbrooke’s Hospital and University of Cambridge School of Clinical Medicine, Cambridge Biomedical Campus, Box 218, Cambridge, CB2 0QQ UK; 2 IMAGO7 Foundation, Pisa, Italy; 3 GE Healthcare, Munich, Germany; 4 Department of Urology, Addenbrooke’s Hospital, Cambridge, UK; 5 Academic Urology Group, Department of Surgery and Oncology, University of Cambridge, Cambridge, UK; 6 Cambridge Urology Translational Research and Clinical Trials Office, University of Cambridge, Cambridge, UK; 7 CamPARI Prostate Cancer Group, Addenbrooke’s Hospital and University of Cambridge, Cambridge, UK

**Keywords:** Cancer, Urology, Medical research, Oncology, Cancer imaging, Urological cancer

## Abstract

Magnetic resonance fingerprinting (MRF) is a rapidly developing fast quantitative mapping technique able to produce multiple property maps with reduced sensitivity to motion. MRF has shown promise in improving the diagnosis of clinically significant prostate cancer but requires further validation as part of a prostate multiparametric (mp) MRI protocol. mpMRI protocol mandates the inclusion of dynamic contrast enhanced (DCE) imaging, known for its significant T_1_ shortening effect. MRF could be used to measure both pre- and post-contrast T_1_ values, but its utility must be assessed. In this proof-of-concept study, we sought to evaluate the variation in MRF T_1_ measurements post gadolinium-based contrast agent (GBCA) injection and the utility of such T_1_ measurements to differentiate peripheral and transition zone tumours from normal prostatic tissue. We found that the T_1_ variation in all tissues increased considerably post-GBCA following the expected significant T_1_ shortening effect, compromising the ability of MRF T_1_ to identify transition zone lesions. We, therefore, recommend performing MRF T_1_ prior to DCE imaging to maintain its benefit for improving detection of both peripheral and transition zone lesions while reducing additional scanning time. Demonstrating the effect of GBCA on MRF T_1_ relaxometry in patients also paves the way for future clinical studies investigating the added value of post-GBCA MRF in PCa, including its dynamic analysis as in DCE-MRF.

## Introduction

Prostate cancer (PCa) is the second commonest male malignancy worldwide with multiparametric (mp) MRI now recommended by major European and American guidelines as the first-line investigation for patients with suspected early stage disease^1–4^. The current Prostate Imaging Reporting and Data System (PI-RADS) guidelines only incorporate qualitative measures for interpretation, however, quantitative metrics have been suggested as a means of reducing the considerable interobserver variation of PI-RADS evaluation, shortening the learning curve of mpMRI, and improving diagnostic performance^5–8^.

Magnetic resonance fingerprinting (MRF) is a quantitative technique able to simultaneously generate multiple inherently spatially registered property maps (e.g. T_1_, T_2_, apparent proton-density). Quantitative mapping provided by MRF has demonstrated high reproducibility between centers over standard T_1_ or T_2_ mapping techniques that can have a system dependence^9,10^. These maps can be obtained in the presence of motion while being acquired within imaging times comparable to or faster than conventional mapping techniques^11,12^. The described features of MRF present a particular interest for cancer imaging, where fast and robust quantitative characterization of tissue biology can add value to routinely used qualitative measures for image assessment^12^.

MRF has shown promise for identifying both peripheral zone (PZ) and transition zone (TZ) prostate lesions, demonstrating added value to standard mpMRI sequences for differentiating between indolent and clinically significant disease^13–15^. Further prospective validation of MRF requires additional evaluation of how it can be incorporated into a standard clinical prostate mpMRI protocol, which includes dynamic contrast enhanced (DCE) imaging^3^. Gadolinium is known to have a preferential T_1_ shortening effect at low doses, which was confirmed for MRF in pre-clinical studies involving a murine glioblastoma model^16,17^. However, no attempts have been made to investigate the impact of gadolinium-based contrast agents (GBCA) on MRF-based T_1_ relaxometry within patients in the clinical setting.

For prostate imaging, understanding the potential added value and robustness of post-GBCA MRF is of considerable practical interest for two major reasons. Firstly, should MRF be incorporated into the clinical mpMRI protocol, the decision on its running order in relation to DCE-MRI should be evidenced and balanced against the increasing trend towards reducing scanning time^18–21^. Secondly, post-GBCA MRF T_1_ mapping may improve the performance of DCE-MRI in TZ lesions, where it is currently of limited use in the context of adequate T_2_-weighted imaging and diffusion-weighted imaging^22,23^. Finally, investigating the robustness of MRF T_1_ relaxometry post GBCA in the clinical setting would also be relevant to cancers located in other anatomical regions where MRF has also shown promising results and the use of GBCA is routine.

Therefore, in this proof-of-concept study we sought to evaluate the variation in MRF T_1_ measurements post GBCA administration and evaluate its impact on the technique’s ability to differentiate between tumor and normal tissue in patients with biopsy-proven PZ and TZ prostate lesions. To ensure the robustness of the MRF technique used, we also validated it against “gold standard” quantitative mapping techniques as part of a phantom study.

## Results

### Phantom results

**Figure 1:**
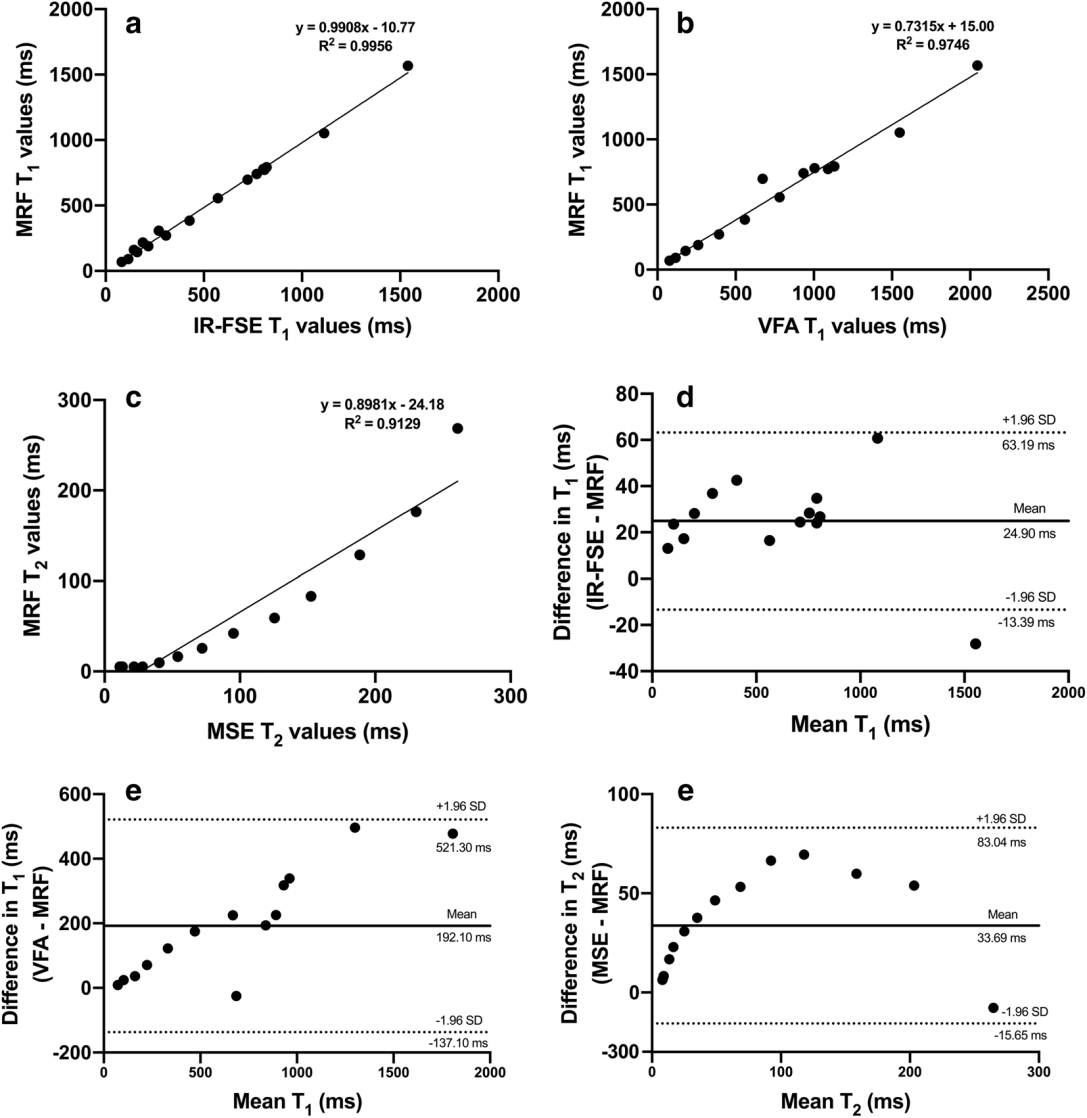
Linear regression plots (**a**–**c**) and Bland–Altman plots (**d**–**f**) comparing MRF T_1_ values with those obtained using IR-FSE (**a**,**d**) and VFA (**b**,**e**) mapping techniques and MRF T_2_ values with those obtained using MSE technique (**c**,**f**). Figures (**a**–**c**) include captions representing slopes of the linear fits, y-intercepts and coefficients of determination (R2). On figures (**d**–**f**), dotted lines represent upper and lower 95% limits of agreement and bold lines represent the mean biases with appropriate captions included. *MRF* magnetic resonance fingerprinting, *IR-FSE* inversion recovery fast spin echo, *VFA* variable flip angle, *MSE* multiple spin echo, *SD* standard deviation.

Figure 1a,b shows the mean MRF T_1_ values obtained from the ISMRM/NIST phantom plotted against T_1_ values obtained using the “gold standard” inversion recovery fast spin echo (IR-FSE) imaging and 3D variable flip angle (VFA) T_1_ mapping. The results show a strong linear correlation between MRF and IR-FSE (R^2^ = 0.996, p ≤ 0.0001, slope = 0.991) and a slightly weaker correlation between MRF and VFA (R^2^ = 0.975, p ≤ 0.0001, slope = 0.732). The comparison of slopes of the linear fits (presented alongside y-intercepts in Fig. 1a,b) suggests better performance of MRF compared to VFA in the phantom setting.

Figure 1c demonstrates the mean phantom MRF T_2_ values plotted against the values obtained using the “gold standard” multiple spin echo (MSE) T_2_ mapping; slopes and y-intercepts are also shown. Although still significant, the correlation between the values is considerably lower than for both T_1_ method comparisons presenting as parabolic rather than linear relationship (R^2^ = 0.9129, p ≤ 0.0001), suggesting lower reliability of MRF T_2_ mapping used in this study.

Bland–Altman plots were constructed to evaluate the agreement between the aforementioned techniques and are presented in Fig. 1d–f. The mean bias and the 95% limits of agreement (LOA) for T_1_ values obtained using IR-FSE and MRF and VFA and MRF are presented in Fig. 1d,e, respectively, while the same parameters for T_2_ values obtained using MSE and MRF are shown in Fig. 1f. One data point with the longest T_1_ was outside the LOA for IR-FSE vs MRF.

### Patient characteristics

The study included 14 patients with biopsy-proven PCa with mean age 70 years (IQR, 67.3–73.5 years), mean PSA 6.29 ng/mL (IQR, 3.8–8.7 ng/mL), with mean time since last biopsy being 16 months (range 4–48 months). A total of 19 MR-visible prostate lesions were included in the analysis, 10 of which were located in the peripheral zone (PZ) and 9 in the transition zone (TZ). Three lesions exhibited intermediate-grade Gleason score 3 + 4 = 7 disease (grade group 2) while other lesions harboured low-grade disease with Gleason score of 3 + 3 = 6 (grade group 1).

### In vivo agreement between MRF-, VFA- and MSE-based T_1_ and T_2_ relaxometry

**Figure 2:**
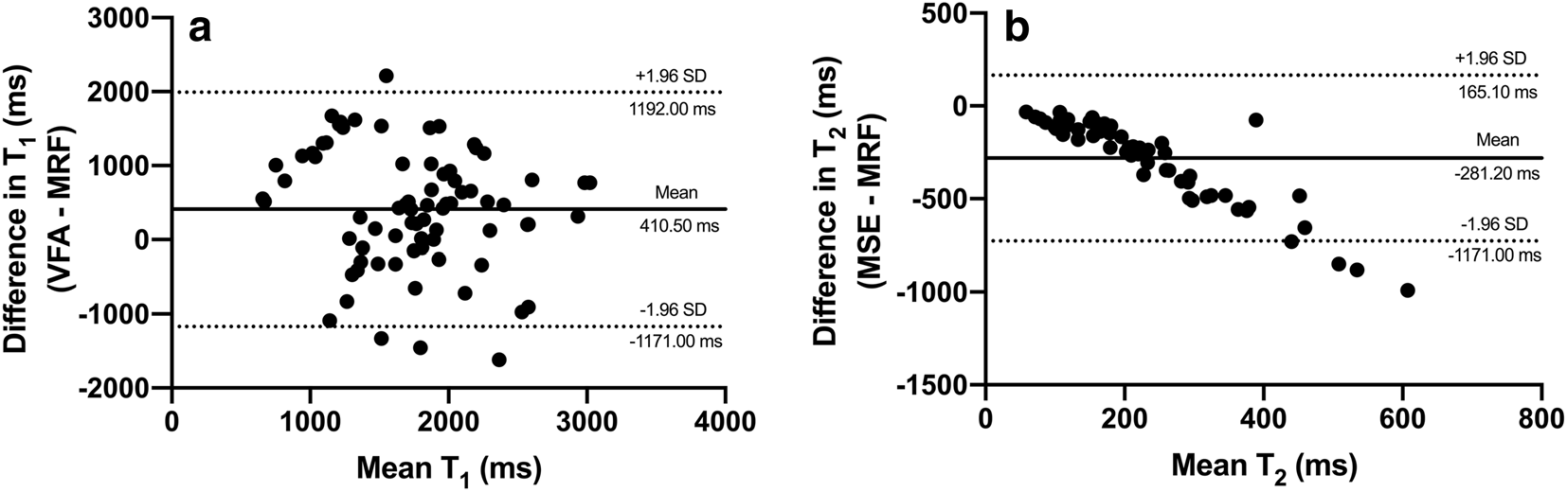
Bland–Altman plots comparing pre-GBCA in vivo MRF and VFA T_1_ values (**a**) and MRF and MSE T_2_ values (**b**) obtained from all tissues included in the analysis. Dotted lines represent upper and lower 95% limits of agreement and bold lines represent the mean biases with appropriate captions included. *MRF* magnetic resonance fingerprinting, *VFA* variable flip angle, *MSE* multiple spin echo.

Summary pre- and post-GBCA MRF- and pre-GBCA VFA- and MSE-based T_1_ and T_2_ along with ADC values obtained from all prostate lesions combined (n = 19), PZ (n = 10) and TZ (n = 9) lesions, pooled nPZ and nTZ, internal obturator muscle and subcutaneous fat (n = 14 for all) are presented in Table 1. Bland–Altman analysis showed lower agreement between pre-GBCA MRF- and VFA-based T_1_ relaxation times compared to the phantom experiment, which is to be expected given the physiological motion in vivo, with the mean bias being 410.5 ms and the 95% LOA ranging between − 1171.0 and 1192.0 ms as demonstrated in Fig. 2a. A similar trend was revealed when the Bland–Altman analysis was used to evaluate the agreement between pre-GBCA MRF- and MSE-based T_2_ relaxation times, with the mean bias being − 281.2 ms and 95% LOA ranging between − 727.5 ms and 165.1 ms (Fig. 2b).

**Table 1:** Summary values derived from different tissue types from MRF T_1_ and T_2_ maps obtained both before and after gadolinium-based contrast agent administration as well as those derived from pre-contrast VFA T_1_, MSE T_2_ and ADC maps.

Tissue	MRF T_1_ (pre-Gd), ms	MRF T_1_ (post-Gd), ms	MRF T_2_ (pre-Gd), ms	MRF T_2_ (post-Gd), ms	VFA T_1_ (pre-Gd), ms	MSE T_2_ (pre-Gd), ms	ADC (pre-Gd), mm^2^/s
All lesions	1666.0 ± 294.0	717.8 ± 346.0	443.6 ± 259.0	252.9 ± 167.5	1990.0 ± 522.7	76.6 ± 27.8	0.94 ± 0.17
PZ lesions	1640.0 ± 368.1	678.4 ± 287.9	507.8 ± 292.7	273.4 ± 160.7	1986.0 ± 629.5	89.2 ± 21.8	0.93 ± 0.16
TZ lesions	1696.0 ± 200.5	761.5 ± 414.8	372.2 ± 209.0	230.1 ± 181.5	2002.0 ± 413.7	69.3 ± 9.8	0.90 ± 0.14
Normal PZ	2521.0 ± 405.9	1270 ± 224.6	546.7 ± 294.0	326.5 ± 255.3	2188.0 ± 813.9	139.4 ± 79.12	1.61 ± 0.22
Normal TZ	1753.0 ± 444.7	723.8 ± 407.3	451.0 ± 228.0	237.9 ± 270.5	2118.0 ± 732.1	88.56 ± 11.67	1.27 ± 0.14
Muscle	1542 ± 211.4	1214 ± 149.4	232.2 ± 156.3	180.6 ± 142.4	1813.0 ± 334.9	41.6 ± 2.7	1.17 ± 0.04
Fat	414.3 ± 67.1	327.4 ± 65.4	240.4 ± 41.1	213.7 ± 41.5	1682.0 ± 491.3	115.7 ± 6.6	0.08 ± 0.07

*MRF* magnetic resonance fingerprinting, *VFA* variable flip angle, *MSE* multiple spin echo, *ADC* apparent diffusion coefficient, *PZ* peripheral zone, *TZ* transition zone.

### MRF T_1_ variation post GBCA

**Figure 3:**
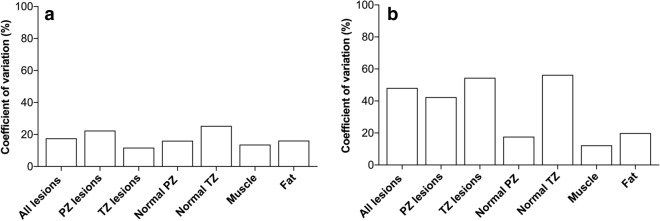
Coefficients of variation of pre-gadolinium (**a**) and post-gadolinium (**b**) MRF T_1_ relaxation times obtained from prostate lesions, pooled normal PZ and TZ, internal obturator muscle and subcutaneous abdominal fat. *PZ* peripheral zone, *TZ* transition zone, *MRF* magnetic resonance fingerprinting.

Table 2 presents coefficients of variation (CVs) calculated for all acquired values to compare their variation in different tissues. Pre-GBCA MRF T_1_ demonstrated low variation in all tissues except for nTZ, where it reached 25.4%. (Fig. 3a) Post-GBCA, MRF T_1_ variation was above 25% in all tissue types except nPZ, muscle and fat, where CVs remained in the same category as pre-GBCA. A particularly marked, almost five-fold increase in data heterogeneity was observed for MRF T_1_ values obtained from TZ lesions and, to a lesser extent nTZ, whereas PZ lesions demonstrated only a two-fold increase in CV and only a marginal change in variation was noted in nPZ. (Fig. 3b; Table 2).

**Table 2:** Coefficients of variations of pre-contrast and post-contrast MRF T_1_ and T_2_ maps as well as pre-contrast VFA T_1_, MSE T_2_ and ADC maps derived from different tissue types.

Tissue	MRF T_1_ (pre-Gd), %	MRF T_1_ (post-Gd), %	MRF T_2_ (pre-Gd), %	MRF T_2_ (post-Gd), %	VFA T_1_ (pre-Gd), %	MSE T_2_ (pre-Gd), %	ADC (pre-Gd), %
All lesions	17.6	48.2	58.4	66.2	26.3	24.0	17.6
PZ lesions	22.5	42.4	57.6	58.8	31.7	24.5	17.5
TZ lesions	11.8	54.5	56.2	78.9	20.7	14.2	15.6
Normal PZ	16.1	17.7	53.8	78.2	37.2	56.7	13.9
Normal TZ	25.4	56.3	50.6	113.7	34.6	13.2	10.9
Muscle	13.7	12.3	67.3	78.9	18.5	6.6	3.7
Fat	16.2	20.0	17.1	19.4	29.2	5.7	8.0

*MRF* magnetic resonance fingerprinting, *VFA* variable flip angle, *MSE* multiple spin echo, *ADC* apparent diffusion coefficient, *PZ* peripheral zone, *TZ* transition zone.

### MRF T_2_ variation post GBCA

In line with the outcomes of the Bland–Altman analysis indicating lower robustness of MRF T_2_ compared to “gold standard” T_2_ mapping, pre-GBCA MRF T_2_ values were considerably more variable compared to pre-GBCA MRF T_1_ values in all tissues except fat, further suggesting low reliability of in vivo MRF T_2_ mapping used in this study. The variation of MRF T_2_ also increased considerably post-GBCA reaching 113.7% in nTZ (Table 2).

### MRF-based T_1_ relaxometry for differentiating tumour and normal tissue

**Figure 4:**
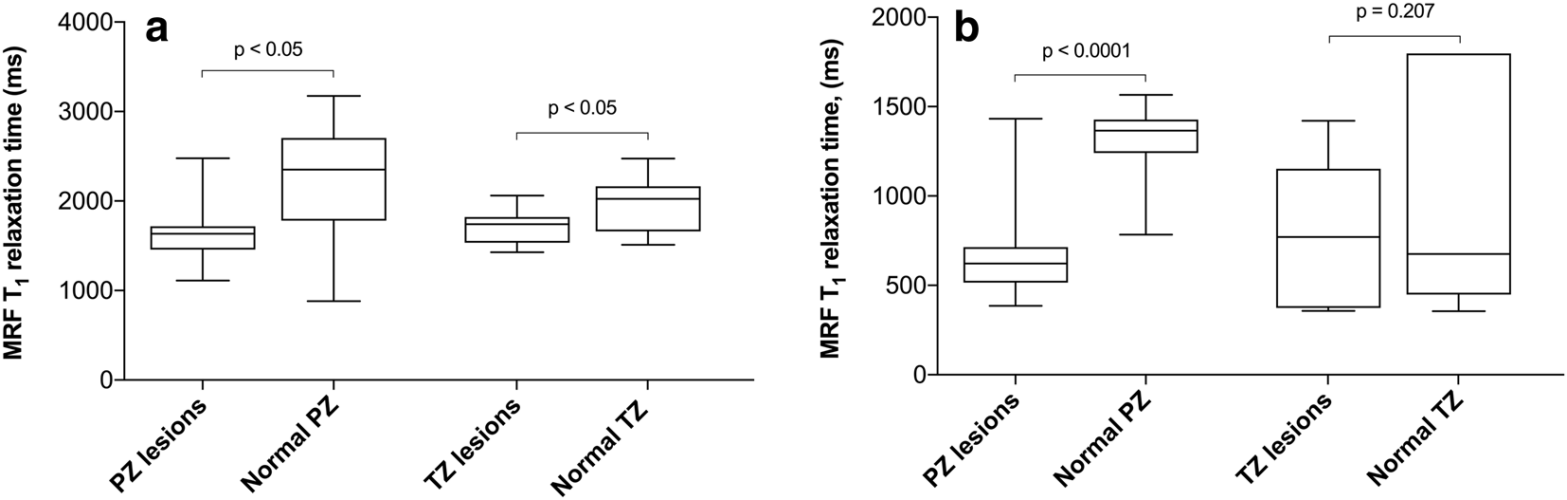
Box-and-whisker plots comparing MRF T_1_ relaxation times obtained from prostate lesions and corresponding normal PZ and TZ before (**a**) and after (**b**) gadolinium-based contrast agent administration. Top and bottom of boxes represent 25th and 75th percentiles of data, respectively; line in boxes represents the median value and bars represent minimum and maximum values. *MRF* magnetic resonance fingerprinting, *PZ* peripheral zone, *TZ* transition zone.

**Figure 5:**
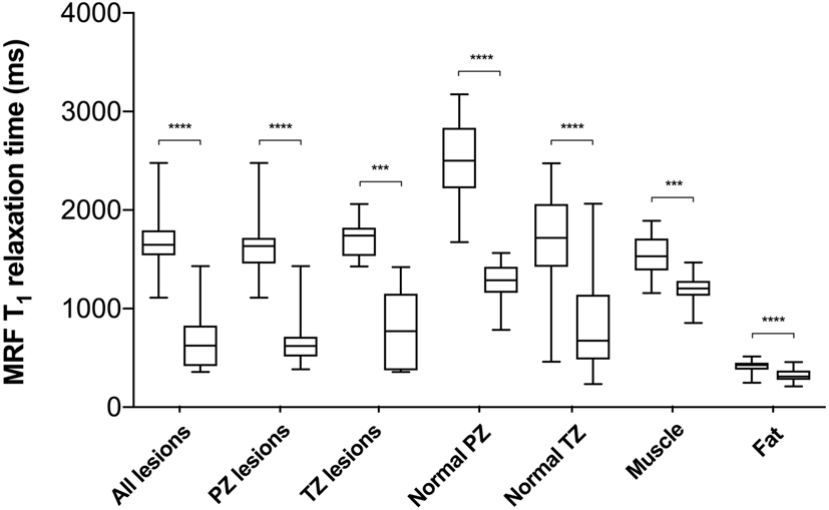
Box-and-whisker plots comparing MRF T_1_ relaxation times obtained before and after gadolinium-based contrast agent administration in prostate lesions, pooled normal PZ and TZ, internal obturator muscle and subcutaneous abdominal fat. *MRF* magnetic resonance fingerprinting, *PZ* peripheral zone, *TZ* transition zone. ****p < 0.0001, ***p = 0.0002.

Prior to GBCA administration, a paired *t* test revealed significantly shorter MRF T_1_ values for both peripheral and transition zone lesions when compared to corresponding nPZ and nTZ in the same patients (1640.0 ms ± 368.1 ms vs 2200.0 ms ± 776.5 ms for PZ and 1696.0 ms ± 200.5 ms vs 1966.0 ms ± 315.1 ms for TZ; p = 0.03 and 0.013, respectively) (Fig. 4a). In pooled nPZ, MRF T_1_ relaxation time was significantly longer than in nTZ (2521.0 ms ± 405.9 ms vs 1753.0 ms ± 444.7 ms; p < 0.0001). Post-GBCA MRF T_1_ remained significantly shorter within peripheral zone lesions compared to the corresponding normal PZ (678.4 ms ± 287.9 ms vs 1317.0 ms ± 219.6 ms; p < 0.0001), however, there was no longer a significant difference in TZ tumours compared to corresponding nTZ (723.8 ms ± 407.3 ms vs 966.4 ms ± 635.5 ms, p = 0.207) (Fig. 4b). Pooled nTZ T_1_ relaxation time was again significantly shorter than those of nPZ (1270 ms ± 224.6 ms vs 723.8 ms ± 407.3 ms; p < 0.0001). Paired t test showed a significant MRF T_1_ shortening effect of GBCA in all tissues (Fig. 5).

The information about the diagnostic utility of MRF- and MSE-based T_2_, VFA-based T_1_ and ADC mapping is provided in the Supplementary Information S1.

## Discussion

This prospective, proof-of-concept study demonstrates the effect of gadolinium-based contrast agent administration on MRF-based T_1_ relaxometry in the clinical setting. We have shown that GBCA considerably increases MRF T_1_ variation in both normal and malignant prostate tissues and compromises its diagnostic utility in the transition zone. To our knowledge, this is the first study reporting both pre- and post-GBCA MRF T_1_ and T_2_ values as well as a combination of MRF-, VFA- and MSE-based T_1_ and T_2_ values obtained from the same patients with prostate cancer. These results will help inform future studies, when MRF may be incorporated into prostate mpMRI protocols as an additional sequence.

The observed MRF T_1_ shortening effect post-GBCA is expected, as gadolinium facilitates both longitudinal and transverse magnetic relaxation, thereby shortening both T_1_ and T_2_ of tissues^24,25^. Other authors also observed a similar trend when measuring gadolinium and dysprosium concentrations in murine glioma models using dual contrast-MRF. ^16,26^ The observed GBCA-induced increase in MRF T_1_ heterogeneity varied between tissues. Normal TZ exhibited the greatest variation pre-GBCA, which is expected in this age group, given the high prevalence of benign prostatic hyperplasia (BPH). Marked hypervascularity within BPH nodules may additionally explain the more marked (two-fold) increase in MRF T_1_ heterogeneity in nTZ tissue following GBCA administration^22^. The finding of increased MRF T_1_ variation was even more marked for TZ lesions (almost five-fold), which likely reflects previously reported large variation in their microvascular parameters and explains the inability of MRF T_1_ to identify TZ lesions post-GBCA^27^. Although a study by Panda et al. has shown the added value of pre-GBCA MRF T2WI, which is considered the primary sequence for assessment of the TZ^28–30^, the reduced performance of post-GBCA MRF T_1_ in assessing TZ lesions should be considered when deciding on its timing in relation to DCE. Conversely, normal PZ, fat and muscle, which can be considered as “control” type tissues given their relatively low vascularity and morphological homogeneity in this age group, maintained low MRF T_1_ heterogeneity post-GBCA. Hence, understanding the rationale for acquiring MRF T_1_ prior to GBCA administration would not only ensure its optimal performance for both TZ and PZ assessment but also enable evidenced planning of the overall scanning time, which is critical due to the growing demand on imaging services.

We also demonstrated that pre-GBCA MRF T_1_ relaxation times were significantly lower in cancers compared to normal tissue in both the TZ and PZ of the prostate. These findings align well with previous studies where a combination of MRF and ADC maps worked best for identifying PZ and TZ lesions; our MRF T_1_ and ADC absolute values are similar to these reported values^13–15^. Our pre-GBCA T_1_ values obtained from PZ and TZ lesions are comparable to those reported previously in clinically significant PCa (1628.0 ms ± 344.0 ms vs 2247.0 ms ± 450 ms for PZ lesions and nPZ; 1450 ms ± 110 ms vs 1800 ms ± 150 ms for TZ lesions and nTZ)^13,14^ and consistent with those reported by Yu et al*.*^15^ for low-grade tumours that dominated the sample size in our study (1679.0 ms ± 422 ms). Although we showed low variation of ADC values, significant motion and susceptibility artefacts were not observed in our cohort, which is less representative of real-life clinical practice where motion often hinders assessment of the peripheral zone^31–33^, for which DWI is the key sequence and where 75–80% of clinically significant lesions are located^34,35^. Acceptable pre-GBCA variation of MRF T_1_ coupled with the technique’s intrinsic robustness to motion therefore further support the need for investigating the added value of MRF in prostate imaging, particularly when DWI fails due to artefact.

As expected, MRF had considerably shorter maximum scanning time of 3 min 40 s compared to standard T_1_ and T_2_ mapping at 4 min 20 s and almost 6 min, respectively. The additional benefit of MRF for prostate imaging is that it can be sensitive to a wider range of T_1_ values (in this work, 1000–2000 ms) than VFA, owing to MRF’s extended range of measured flip angles. Conventional T_1_ mapping techniques, in turn, can be confounded by T_2_ effects, the choice of flip angles, and field (B_1_+) non-uniformity^36–38^. This reduction of T_1_ sensitivity to the aforementioned confounders is illustrated by our lower measured heterogeneity for all tissue types when compared with VFA T_1_ measurements, which may explain superior performance of pre-GBCA MRF T_1_ in vivo for differentiating tumour versus normal prostatic tissue in both PZ and TZ; better performance of MRF T_1_ was also demonstrated in the phantom study. Lower heterogeneity was noted for MSE T_2_ mapping, which is more well-studied in prostate cancer^39^.

This study has several limitations. Firstly, the small sample size may have artificially increased data variation leading to the inability of post-GBCA MRF T_1_ and pre-GBCA VFA T_1_ and MSE T_2_ to identify TZ and PZ tumours, respectively, and did not allow us to quantify grade-dependent variation of MRF T_1_ post GBCA. Secondly, only patients with low- and intermediate-grade disease were included in this study, which may also have had an impact on the diagnostic utility of both MRF and conventional mapping techniques. However, the inability of post-GBCA MRF T_1_ to detect any TZ lesions regardless of their Gleason grade indicates a reduction in robustness of the technique, while the added value of pre-GBCA MRF in detecting both TZ and PZ lesions has been demonstrated previously. VFA- and MSE-based T_1_ and T_2_ maps were not acquired post-GBCA, however, a head-to-head comparison of post-GBCA MRF and standard mapping techniques was not the purpose of this study. In vivo MRF-derived T_2_ values were unreliable in this study showing parabolic relationship with MSE-based T_2_ values, the reliability of which was also undermined by the longest TE being four-fold shorter than the calculated T_2_. However, gadolinium is known to have a more prominent T_1_ shortening effect at low doses, which underpinned the focus of this work on MRF T_1_ relaxometry. Moreover, as evidenced by the comparison with the “gold standard” IR-FSE, MRF T_1_ mapping used in this study was considered robust. In future work investigating T_2_ with MRF, we would give strong consideration for methods that increase the signal-to-noise ratio by increasing the slice thickness, voxel sizes, or by performing averaging with additional acquisitions. While averaging for MRF T_2_ mapping would remain sensitive to motion, we hypothesize that this effect would be reduced due to the pattern matching algorithm of MRF, therefore, supporting the clinical feasibility of both MRF T_1_ and T_2_ mapping. Acquiring MSE-based T_2_ maps with longer TEs to match the known T_2_ values observed in the prostate could also be helpful to improve their reliability and address the reported parabolic relationship with MRF, which may, however, represent a consistent trend worth further investigation.

In conclusion, GBCA administration leads to a considerable increase in MRF T_1_ variation following the expected significant T_1_ shortening effect and compromises its ability to detect TZ lesions. Therefore, performing MRF T_1_ prior to DCE imaging as part of a prostate mpMRI protocol should be considered as a preferred option to retain the technique’s added value for both PZ and TZ lesions and reduce the additional scanning time.

## Methods

### Phantom study

To evaluate the accuracy of T_1_ and T_2_ measurements, MRF and standard relaxation mapping data were obtained from the ISMRM/NIST phantom^40^. Phantom data were obtained on a 3 T MR750 scanner (GE Healthcare, Waukesha, WI, USA) using a 32-channel receiver coil. Regions-of-interest (ROI) were created from the spheres in either the T_1_ or T_2_ layer of the phantom.

Conventional T_1_ maps were obtained with an inversion recovery (IR) and variable flip angle (VFA) techniques. A conventional T_2_ map was obtained with multiple spin echo (MSE) measurements. The field-of-view (FOV) = 260 × 260 mm2, matrix = 256 × 256 and slice thickness = 3 mm matched in all techniques. Inversion recovery fast spin echo (IR-FSE) T_1_ maps from the T_1_ layer were obtained with inversion times (TI) = 50 ms, 100 ms, 200 ms, 400 ms, 800 ms, 1600 ms, 2400 ms, repetition time (TR) = 8000 ms and echo time (TE) = 13 ms. Multiple 3D GRE sequences were used for T_1_ mapping using the VFA method with flip angles of 2°, 5°, 8°, 12°, 15°, 18°, 22°, 26°, TR = 10 ms, TE = 1.6 ms. MSE T_2_ maps were obtained with TR = 600 ms and TEs = 8.2, 16.3, 24.5, 32.6, 40.8, 49.0, 57.1, 65.3 ms. Fitting was performed using a non-linear least squares fit to the IR, VFA and MSE signal equations in Python.

MR fingerprinting was also performed as further described in the *MR fingerprinting protocol* subsection.

### Patient study

All elements of this prospective study were carried out in accordance with the Declaration of Helsinki and were approved by the institutional ethics board (NRES Committee East of England, UK), with written informed consent obtained from all participants. All methods were performed in accordance with the relevant guidelines and regulations. Patients on active surveillance, with MR-visible, biopsy-proven prostate cancer were included in this study. Exclusion criteria included prostate biopsy within the preceding 3 months, presence of pelvic metalwork, or any previous treatment for PCa.

### Biopsy technique

Depending on clinical recommendation, biopsy was performed by either a transrectal or transperineal approach, using MRI/ultrasound fusion. All biopsy procedures were performed by experienced urologists and included 12–24 systematic cores, with 2–4 separate target cores acquired from the MRI defined lesion/s. All targets were defined by radiologists pre-procedure using T2-weighted imaging as the primary and diffusion-weighted imaging as the secondary source images, using the DynaCAD system (InVivo Corp, Orlando, FL) for transrectal and Biopsee software (Oncology Systems Limited, Shrewsbury, UK) for transperineal approaches as previously described^41^.

### Multiparametric MRI

Patients underwent prostate MRI on a 3 T MR750 scanner (GE Healthcare, Waukesha, WI, USA) using a 32-channel receiver coil. Intravenous injection of hyoscine butylbromide (Buscopan, 20 mg/mL; Boehringer, Ingelheim am Rhein, Germany) was administered prior to imaging to reduce peristaltic movement, unless clinically contraindicated. Multiparametric MRI protocol included Axial T_1_ and multiplanar high-resolution T_2_-weighted 2D fast recovery FSE (field of view (FOV) 18 × 18 cm; voxel size 0.35 × 0.35 mm^2^; slice thickness 3 mm; gap 0 mm). Diffusion-weighted imaging (DWI) was performed using a spin-echo echo-planar imaging pulse sequence (FOV 28 cm; slice thickness 3 mm; gap 0 mm; b-values: b-150, b-750, and b-1400 s/mm^2^) and an additional small FOV (24 cm) b-2000 s/mm^2^ DWI sequence; apparent diffusion coefficient (ADC) maps were calculated automatically. T_1_ and T_2_ mapping were performed with VFA, MSE and MRF prior to dynamic contrast enhancement (DCE). DCE was performed using a standard sequence (FOV 24 cm; slice thickness and gap 3 mm and 0 mm, respectively; temporal resolution 7 s) following a bolus of Gadobutrol (Gadovist, 0.1 mmol/kg, Bayer) at 28 s via a power injector, at a rate of 3 ml/s (dose 0.1 mmol/kg). Post-GBCA MRF was performed immediately after DCE in all patients.

### In vivo variable flip angle (VFA) T_1_ mapping

Multiple 3D GRE sequences were used for T_1_ mapping using the variable flip angle method with flip angles of 2°, 5°, 12°, 20°, and 32°. Each flip angle was acquired in 52 s, for a total duration of 4 min 20 s. Other parameters included: FOV = 36 cm, matrix = 256 × 256, slices = 52, slice thickness = 3 mm, echo time (TE) = 2.0 ms, repetition time (TR) = 15 ms, with 70% sampling.

### In vivo multiple spin echo (MSE) T_2_ mapping

Multiple echo 2D FSE images were acquired for T_2_ mapping; FOV 36 cm, matrix 256 × 256, voxel size 1.4 × 1.4 mm^2^, slice thickness 2.5 mm, TR 2.6 s, TEs = 8.5 ms, 16.9 ms, 25.4 ms, 42.3 ms, 50.8 ms, 59.2 ms, 67.7 ms, averages = 0.5 (partial k-space), acquisition time 359 s (5 min 59 s).

### MR fingerprinting protocol

A 2D steady-state-free-precession (SSFP) MRF sequence with inversion preparation was used for T_1_ and T_2_ mapping with 979 under-sampled, interleaved spirals for *k*-space sampling. The maximum gradient strength per spiral was 28 mT/m and the maximum slew rate was 108 T/m/s. The imaging parameters were: FOV = 260 × 260 mm^2^, matrix = 256 × 256, slices = 15–22, slice thickness = 3.0 mm, spacing 1.0 mm, sampling bandwidth =  ± 250 kHz, slice dephasing = 8π, echo time (TE) = 2.5 ms, repetition time (TR) = 10 ms, acquisition time = 9.79 s/slice (maximum scanning time 3 min 40 s). We used similar flip angle lists to those used in previous works in order to more accurately assess the utility of MRF with standard lists^36,42^. Axial images were acquired to match the standard acquisition planes for other prostate image assessments. MRF image reconstruction, dictionary simulation and pattern matching parameters are listed in the Supplementary information S1.

### Image analysis

MRF T_1_ values for MR-visible lesions (PI-RADS scores 4/5)^6^, normal peripheral zone (nPZ), normal transition zone (nTZ), subcutaneous abdominal fat and normal internal obturator muscle were calculated from ROIs originally drawn on anatomical T_2_-weighted images with reference to ADC maps (as presented in Supplementary Fig. S1) by a single fellowship-trained uro-radiologist with 12 years’ experience of reporting prostate MRI using the open-source segmentation software ITK-SNAP^43^. ROIs were then transposed on to the MRF T_1_ and T_2_, VFA- and MSE-based T_1_ and T_2_ and ADC maps with their size and location being matched to the appropriate FOV parameters and anatomical position of the outlined structures using in-house software developed within Python using the PyQtGraph and PyDicom libraries^44^. nPZ and nTZ ROIs were drawn in regions with biopsy-confirmed healthy tissue.

### Statistics

In the phantom study, simple linear regression was used to evaluate the relationship between T_1_ and T_2_ values obtained using IR-FSE, VFA, MSE and MRF mapping techniques with their agreement assessed using the Bland–Altman analysis. In the patient study, the Shapiro–Wilk test was applied to assess the normality of imaging values with their intergroup comparison performed using either paired or unpaired *t* test as appropriate. Planned independent paired comparisons between MRF T_1_ and T_2_ values obtained from PZ and TZ lesions versus corresponding nPZ and nTZ were not adjusted for multiplicity; all other post hoc comparisons were adjusted using the Holm–Šidak method with alpha set at 0.05 as advised by an expert biostatistician. The variation of imaging values was evaluated using coefficient of variation (CV); CV of less than 25% indicated acceptable heterogeneity. Agreement between the conventional VFA- and MRF-based T_1_ relaxation times, treated as single paired measurements, was assessed using the Bland–Altman analysis. All plots and figures were created in Prism 8 (GraphPad Software, San Diego, CA, USA).

## Supplementary information

Supplementary Information.

## Data Availability

The primary research data is available at https://data.mendeley.com/datasets/g3k7xwjpd3/draft?a=da4b46df-b5b3-4dac-aa8d-84b8c75ee526 (10.17632/g3k7xwjpd3.1).
