# Supplementary material for: The effect of gadolinium-based contrast agent administration on magnetic resonance fingerprinting-based T1 relaxometry in patients with prostate cancer
Source: Sci Rep. 2020 Nov 24;10:20475. doi: 10.1038/s41598-020-77331-4 (PMC7686305; doi:10.1038/s41598-020-77331-4)
Supplement: Supplementary file 1 — Supplementary Information. [file 41598_2020_77331_MOESM1_ESM.pdf]

The effect of gadolinium-based contrast agent administration on magnetic resonance fingerprinting-based T_1 relaxometry in patients with prostate cancer

Nikita Sushentsev[1†], MD, Joshua D Kaggie[1†], PhD, Guido Buonincontri[2], PhD, Rolf F Schulte[3], PhD, Martin J Graves[1], PhD, Vincent J Gnanapragasam[4,5,6], PhD, Tristan Barrett[1,7*], MD

Supplementary information.

Methods.

MRF image reconstruction

Each under-sampled spiral was reconstructed, re-gridded and reconstructed to image space. After reconstruction, each coil channel was combined using adaptive coil combination based on weights determined from the average of the time frames[1]. The under-sampled images were reduced from 979 to 16 images using the singular value decomposition (SVD) weights determined during dictionary compression[2].

MRF dictionary simulation

Dictionary was simulated using the extended phase graph formalism, and included the slice profile[3]. The ranges and incremental (step-size) changes of the T1 and T2 values that were simulated in the dictionary were T_1 = [0.01:0.005:1; 1.04:0.04:6] seconds ([minimum: step-size: maximum]), and for T_2 = [0.005:0.001:0.1; 0.1:0.01:4; 4:0.04:6] seconds (where the semi-colons indicate concatenated lists). The dictionary size was compressed to 16 singular vectors (rank) with SVD to reduce the size for long term storage and faster dictionary matching[2].

MRF pattern matching

T_1 and T_2 values are were assigned to each voxel after pattern matching. MRF used inner product pattern matching of the signals with the simulated dictionary to obtain the best T_1 and T_2 match with the acquired reconstructed data. The inner products between the normalized measured signal evolution of each voxel and each normalised dictionary entry were calculated. The dictionary entry returning the maximum value for the inner product was taken as the best representation of the acquired signal evolution.

Results.

Pre-gadolinium VFA-based T_1, MSE-based T_2 and ADC mapping ability to differentiate tumour and normal tissue

VFA-based T_1 relaxation times showed proportionately higher heterogeneity compared to MRF T_1. **(Table 2)** VFA-based T_1 relaxation times did not differ between PZ and TZ lesions and corresponding nPZ and NTZ (1986.0 ms ± 629.5 ms vs 2172.0 ms ± 779.3 ms for PZ and nPZ; 2002.0 ms ± 413.7 ms vs 2341 ms ± 532.9 ms for TZ and nTZ; p = 0.666 and 0.199, respectively). No difference in VFA-based T_1 was noted between pooled nPZ and nTZ (2188.0 ms ± 813.9 ms vs 2118.0 ms ± 732.1 ms; p = 0.930).

Similar to T_1, MSE-based T_2 relaxation times did not differ between PZ lesions and corresponding nPZ (89.2 ms ± 21.8 ms vs 144.0 ms ± 93.6 ms for T_2; p = 0.566), however being significantly shorter in TZ lesions compared to normal tissue (69.3 ms ± 9.8 ms vs 92.4 ms ± 14.1 ms for T_2; p= 0.035). T_2 values of nPZ were similar to those of nTZ (139.4 ms ± 79.12 ms vs 88.56 ms ± 11.67 ms; p = 0.369).

ADC values were significantly lower in both PZ and TZ lesions compared to corresponding nPZ and nTZ (0.93 ± 0.16mm^2/s vs 1.61 ± 0.22mm^2/s for PZ and 0.90 ± 0.14mm^2/s vs 1.35 ± 0.15 mm^2/s for TZ; p = 0.0015 for both). Pooled nTZ had lower ADC values than nPZ (1.27 mm^2/s ± 0.14 mm^2/s vs 1.61 mm^2/s ± 0.22 mm^2/s; p = 0.0052).

MRF-based T_2 mapping for differentiating tumour and normal tissue

Pre- GBCA MRF T_2 relaxation times were shorter in tumours compared to corresponding nPZ and nTZ (507.8 ms ± 292.7 ms vs 527.7 ms ± 255.4 ms for PZ and 372.2 ms ± 209.0 ms vs 527.1 ms ± 246.4 ms for TZ; p = 0.960 and 0.666, respectively), however, the results lacked statistical significance. No difference was also observed between MRF-based T_2 of pooled nPZ and nTZ (546.7 ms ± 294.0 ms vs 451.0 ms ± 228.0 ms; p = 0.930). Post- GBCA MRF T_2 values were slightly shorter in lesions compared to corresponding normal zones, but this difference was not significant (273.4 ms ± 160.7 ms vs 326.3 ms ± 269.9 ms for PZ and 230.1 ms ± 181.5 ms vs 113.3 ms ± 37.2 ms for TZ; p = 0.960 and 0.485, respectively). There was also no difference between MRF T_2 relaxation times of pooled nPZ and nTZ (326.5 ms ± 255.3 ms vs 237.9 ms ± 270.5 ms, p= 0.930).

Supplementary Figure 1.

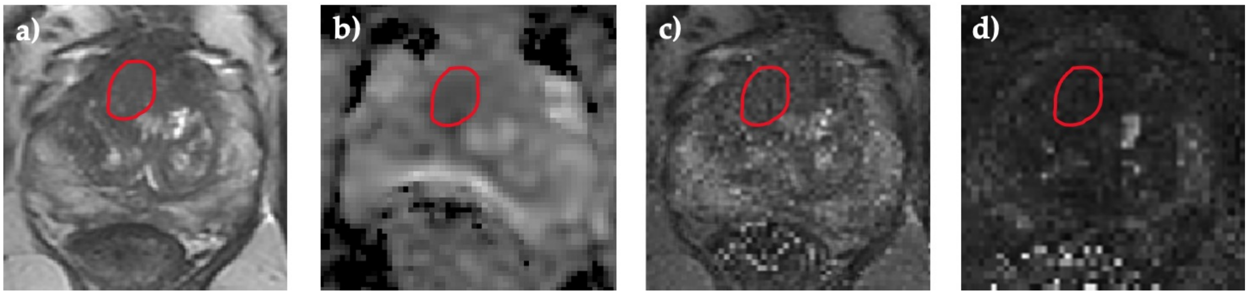

Supplementary Figure 1. Sample T2-weighted image (a), ADC map (b) and MRF-derived T1 pre-contrast (c) and post-contrast (d) maps obtained from a 63-year old male with an outlined anterior midline transition zone prostate lesion (Gleason score 3+3=6). The original region-of-interest was drawn on T2-weighted image being subsequently transposed onto registered ADC and MRF maps as described in the manuscript. A visible decrease in image contrast is noted on post-gadolinium image d). Window width and window level are the same for images c) and d).

References:

1. DO, W., AF, G. & MW, M. Adaptive Reconstruction of Phased Array MR Imagery. *Magn. Reson. Med.* **43**, (2000).

2. McGivney, D. F. *et al.* SVD compression for magnetic resonance fingerprinting in the time domain. *IEEE Trans. Med. Imaging* **33**, 2311–2322 (2014).

3. Hennig, J., Weigel, M. & Scheffler, K. Calculation of Flip Angles for Echo Trains with Predefined Amplitudes with the Extended Phase Graph (EPG)-Algorithm: Principles and Applications to Hyperecho and TRAPS Sequences. *Magn. Reson. Med.* **51**, 68–80 (2004).
